# Characteristics of Psychiatric Emergency Situations and the Decision-Making Process Leading to Involuntary Admission

**DOI:** 10.3389/fpsyt.2018.00760

**Published:** 2019-01-18

**Authors:** Silvan Marty, Matthias Jaeger, Sonja Moetteli, Anastasia Theodoridou, Erich Seifritz, Florian Hotzy

**Affiliations:** ^1^Faculty of Medicine, University of Zurich, Zurich, Switzerland; ^2^Department of Psychiatry, Psychotherapy and Psychosomatics, University Hospital of Psychiatry Zurich, Zurich, Switzerland; ^3^Psychiatrie Baselland, Liestal, Switzerland

**Keywords:** involuntary admission, psychiatric emergency situation, coercion, decision-making, referring physician

## Abstract

**Introduction:** Involuntary admissions to psychiatric hospitals, regardless of their beneficial effects, violate the patients' autonomy. To keep such measures at a minimum and develop less restricting and coercive alternatives, a better understanding of the psychiatric emergency situations which end up in involuntary admissions is needed. This descriptive and exploratory study investigates the consultations leading to involuntary admission and the decision-making process of the referring physicians.

**Methods:** We developed an online questionnaire to collect data on the characteristics of the consultation leading to an involuntary admission, including influencing factors from the referring physicians‘ perspective, as well as their professional background. We included 107 physicians who completed the questionnaire after they had referred patients for involuntary admission to one major psychiatric hospital in Switzerland.

**Results:** The referring physicians were heterogeneous regarding their medical background and experience with psychiatric emergency situations. The consultations were time consuming and took place in various locations. Clinical findings, third-party anamnesis and a known psychiatric diagnosis contributed strongest to the decision to admit involuntarily. “Protection from danger to self” was named most frequently as purpose of the admission.

**Discussion:** This study emphasizes the variety of psychiatric emergency situations leading to involuntary admissions. In most cases, several parties are involved and influence the decision together with medical and social factors. To reduce the number of involuntary admissions, alternatives for patients with a high symptom load and at risk of harming themselves are needed. Possible approaches to achieve that reduction and recommendations for further research are provided.

## Introduction

Coercive measures such as involuntary admission (IA) to a psychiatric hospital are commonly used in psychiatric emergency situations (PES) when treatment for a refusing patient seems to be necessary, usually due to a potential danger to the patient or to others in combination with an underlying psychiatric disorder (1, 2). Legal, ethical and medical factors are relevant in the implementation and regulation of such measures. Despite these regulations, IAs violate the patients' rights of freedom and self-determination. Therefore, perceived coercion among patients can be high (3, 4). Some may experience feelings of humiliation and, compared to voluntarily admitted patients, many are less satisfied with the received treatment (5–7). Even retrospectively, a substantial percentage of involuntary admitted patients do not consider their admission as justified (8–10). Therefore, coercive measures have been under discussion in psychiatry for centuries (11, 12). However, among mental healthcare professionals it is widely accepted that IA can be beneficial under certain circumstances (13, 14), and studies have shown little to no differences regarding clinical outcome domains and treatment adherence compared to voluntarily admitted patients (4, 5, 15, 16).

The cross-national variations in this highly sensitive and controversial area are remarkable, with rates of involuntary admission differing enormously across the world (1, 2, 17, 18). Even among different regions within the same country or state (and consequently comparable legal regulations), rather impressive differences exist (19, 20). Considering this background, it seems plausible that other factors than the legal prerequisites—such as mental health service structure, local traditions and policies—play an important role as well (21–24). Therefore, an effort has been put into stimulating and harmonizing research and legal prerequisites in different European countries as well as worldwide in order to develop common guidelines or standards of good practice—with the aim of keeping coercive measures at a minimum (25).

In most countries, a physician is legally enabled to mandate an IA of a patient (17, 26). As the gatekeeper to IA, the physician has a crucial role in implementing legal regulations (27) and weighing risks and benefits of involuntary care for the individual patient (28, 29). Studies indicate that the referring physician's experience or competence with psychiatric emergency situations may be associated with disallowance rate and time to discharge (30–32). It has been shown that referring general practitioners find it difficult to apply the legal criteria and assess the necessity for involuntary care (33, 34). In an Australian study (35), patients detained to an emergency department by decision of ambulance officers had 3 times lower odds of a subsequent involuntary admission to a mental health clinic compared to those detained by physicians. Also, differences regarding the compliance rate with legal requirements and the quality of the commitment certificates among various groups of referring physicians have been shown (36–38).

In Switzerland, a federal republic with 26 cantons (states), IA is regulated both on a national and cantonal level. Criteria for IA are defined in the Swiss Civil Code [Art. 426 (39)], whereas different cantonal laws assign the responsible agents. In the canton of Zurich, every physician can admit a patient involuntarily, while in other cantons that decision is assigned only to a selected group of physicians.

There is limited literature describing the course of the PES and the decision-making process leading to IA from the referring physicians' perspectives. Some studies analyzed who initiated the IA (40, 41). Others investigated reasons for the IA and found that patient's aggressiveness, risk of harm to self or others, discontinuation/reinstatement of medical treatment and various other reasons were named with different frequency and importance depending on the setting of the PES and the referring agent (40–43). However, little is known about factors influencing the decision and the course of the processes, and, to our knowledge, no study analyzed in detail the consultation which led to the consequent IA.

This descriptive and exploratory study intended to investigate the process which leads to IA in the canton of Zurich. We aimed to (1) collect data on the referring physicians‘ professional background, (2) describe the characteristics of the PES leading to IA, and (3) shed light on the process of decision-making and factors influencing it.

## Materials and Methods

### Sample

The University Hospital of Psychiatry Zurich (PUK) with its 320 beds constitutes one of the largest hospitals for adult psychiatry within the region and in all of Switzerland. Its catchment area of about 500,000 residents contains both urban and rather rural regions. To investigate the above-mentioned aspects of IA, the structure of the mental health care system in and around Zurich provides a suitable setting because of its various groups of mental health care providers with their diverse backgrounds.

**Figure 1:**
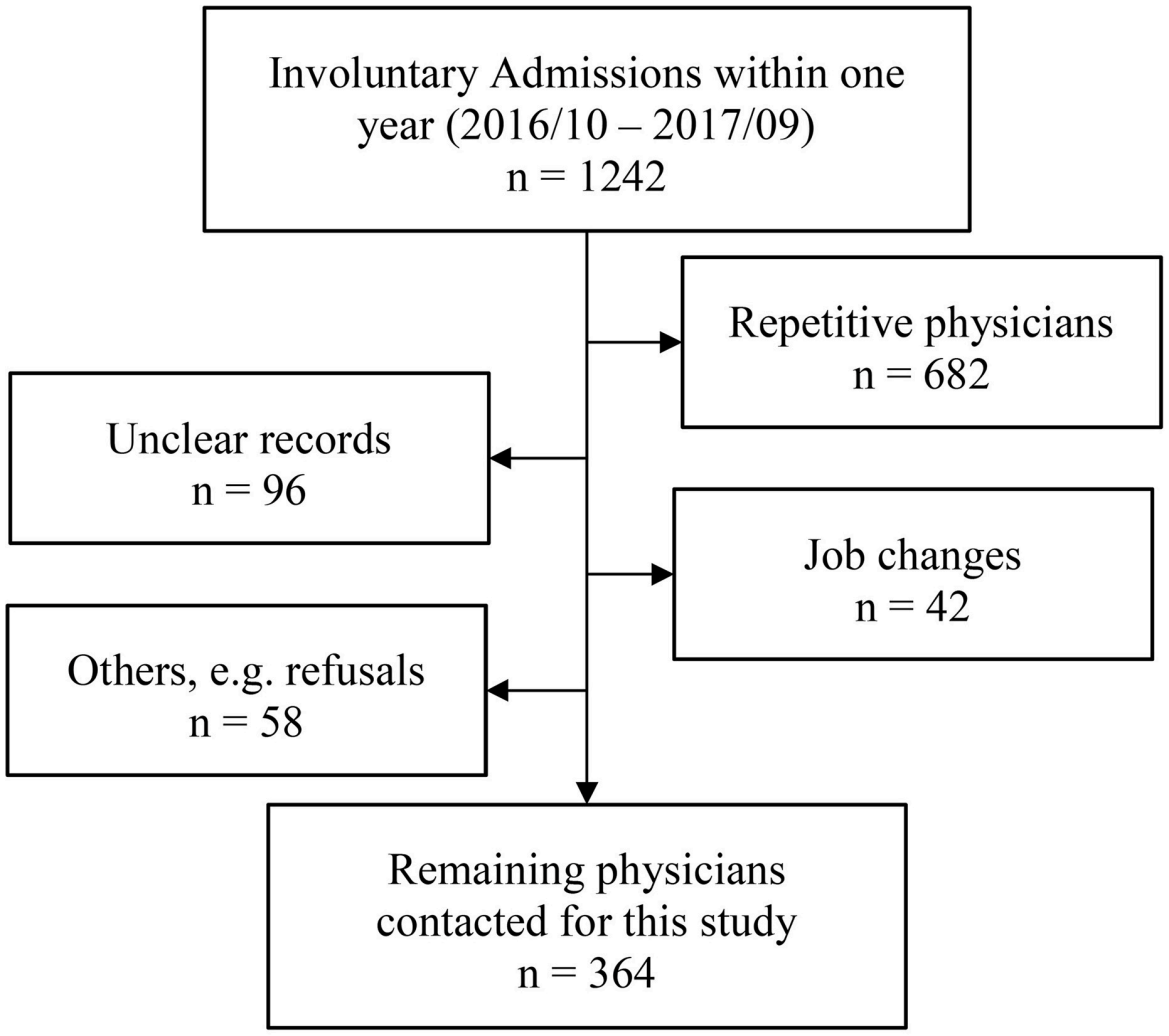
Selection of referring physicians.

We invited all physicians who had referred patients involuntarily to the PUK within a period of 12 months (October 2016–September 2017) to participate in this study. Of the 1,242 records, 682 were repetitions—the same physician referred multiple patients during the period. As shown in Figure 1, 196 records could not be used for other reasons, namely because of missing or unclear contact information, job changes (physicians were not tracked down if they no longer worked at the institution from where the patient was referred), exclusion due to admissions from another canton of Switzerland, or in some cases because the physicians refused to participate. The remaining 364 physicians were contacted by email and invited to answer the questionnaire. Of 109 participants who followed the invitation and completed the questionnaire, two participants had to be excluded due to missing values >50%. Thus, analyses were conducted with 107 (29%) participants.

### Questionnaire

For this study, a structured online questionnaire was developed. The first part of the questionnaire consisted of questions about the physicians' professional background and their experience with PES. In the second part of the questionnaire, we asked physicians some questions on their last PES that led to an IA. This part assessed characteristics of that PES as well as the corresponding decision-making process. The questionnaire was reviewed and discussed by physicians experienced in PES and the referral via IA.

### Subgroups

To compare statements on questions about consultations with colleagues and the use of risk assessment tools we built three subgroups of participants by level of training and medical specialty: (1) Psychiatrist (including child and adolescent psychiatry), (2) Senior doctors who have completed their training with a degree in any other medical specialty, and (3) Residents of any other specialty who have not yet completed their training. The group of referring psychiatrists contained only 5 residents whereof only 1 had <6 years of working experience. Therefore, we decided not to split up that group in seniors and residents for analysis.

### Statistical Analysis

We performed statistical analysis using IBM SPSS Statistics 25 (IBM Corp. Released 2017. IBM SPSS Statistics for Windows, Version 25.0. Armonk, NY: IBM Corp.) and chose a significance level of 0.05. Along with descriptive statistics, we used cross-tables and Chi-Square tests. For Chi-square tests, we reported Fisher's exact test when cell counts <5 were expected. We reported standardized residuals in cross-tables for variables with more than 2 categories.

### Ethics

This study is not subject to the Swiss Human Research Act (Humanforschungsgesetz); therefore, approval from the Cantonal Committee for Ethics was not required. We identified the contacted physicians without collecting any information that would allow conclusions on patients. Furthermore, all data resulting from the online questionnaire have been collected completely anonymously and do not allow to identify neither patient nor referring physician.

## Results

### Participants

Table 1 shows the participants' socio-demographic data as well as data on their professional background and their experiences with PES within the last 12 months. The participants had a mean age of 46.2 years and a mean professional experience of 17.5 years. Nearly half of the participants were psychiatrists working in their own office or in an institution. For 96 (90%) participants, the last time they mandated an IA was no longer than 6 months ago. While mandating the IA, 49 (46%) participants were working in some form of emergency service, whereas the others were working in their regular shifts.

**Table 1:** Participants' socio-demographic data and professional background.

**Characteristics**	***n* (%)^**a**^**
**GENDER**^**b**^	
Female	41 (39)
Male	65 (61)
**AGE**	
<30 years	11 (10)
30–39 years	24 (22)
40–49 years	22 (21)
50–59 years	35 (33)
≥60 years	15 (14)
**PROFESSIONAL EXPERIENCE**^**c**^	
0–2 years	9 (9)
3–5 years	17 (16)
6–10 years	8 (8)
11–20 years	24 (23)
>20 years	47 (45)
**MAIN FIELD OF WORK**	
Outpatient psychiatric office	33 (31)
Psychiatric institution	11 (10)
General practitioner	12 (11)
Outpatient emergency doctor	11 (10)
Hospital–EU	15 (14)
Hospital–not EU	17 (16)
Other	8 (7)
**MEDICAL SPECIALTY**	
Resident psychiatry	5 (5)
Senior doctor psychiatry	42 (39)
Resident internal medicine	21 (20)
Senior doctor internal medicine	29 (27)
Resident other specialty	5 (5)
Senior doctor other specialty	5 (5)
**IAS WITHIN LAST 12 MONTHS**	
1 IA	15 (14)
2–5 IAs	47 (44)
6–10 IAs	29 (27)
>10 IAs	16 (15)

EU, emergency unit, IA, involuntary admission.

a 107 participants, single choice.

b 1 missing

c *2 missing*.

### Characteristics of PES Leading to IA

Table 2 shows different characteristics of the PES leading to IA. The great majority (72%) of the consultations took more than 1 h. Most of the consultations took place in a medical environment, followed by the patient‘s home, the police station and others. Employees of the healthcare system initiated the consultation in most cases, followed by the police, the next of kin and the patients themselves. In about half of the consultations, the police or a security service was involved. In 46%, this involvement was initiated by the referring physicians. Only 3 participants were either alone with the patient or did not answer the question if other people were involved. The use of informal coercion was reported in 54 (50%) cases. Amongst those who had used informal coercion, 42 reported to have done so knowingly, whereas 9 did so unknowingly, and 3 did not answer the question. The use of formal coercion other than IA was less frequent with 27 (25%) reporting the use of some kind of formal coercion.

**Table 2:** Characteristics of PES.

**Characteristics**	***n* (%)**
**DURATION OF CONSULTATION**^**a**^	
<15 min	1 (1)
15–30 min	5 (5)
31–60 min	24 (22)
61–120 min	54 (50)
>120 min	23 (21)
**LOCATION OF CONSULTATION**^**a**^	
Patient's home	27 (25)
Hospital–EU	24 (22)
Hospital–inpatient wards	13 (12)
Police station	11 (10)
Doctor's office	10 (9)
Nursing home	6 (6)
Public space	4 (4)
Other locations	12 (11)
**INITIATING PARTY**^**b**^	
Police	35 (33)
Patient's next of kin / friends	32 (30)
Participant (physician) themself	28 (26)
Nurse	18 (17)
Patient themself	14 (13)
Treating physician	13 (12)
Others	22 (21)
**INVOLVED PARTIES**^**c**^	
Police or security service	53 (50)
Patient's next of kin / friends	50 (47)
Medical rescue service	31 (29)
Nurse	30 (28)
Other physicians	11 (10)
Caregiver sheltered housing	7 (7)
Others	17 (16)
**USE OF INFORMAL COERCION**^**d**^	
None	51 (49)
Persuasion	36 (34)
Negotiation	36 (34)
Pressure	12 (11)
Inducement	10 (10)
Threat	1 (1)
**USE OF FORMAL COERCION**^**e**^	
None	79 (75)
Physical restraint	13 (12)
Police escort	8 (8)
Coercive medication	3 (3)
Others	7 (7)

PES, psychiatric emergency situation, EU, emergency unit.

a 107 participants, single choice.

b 107 participants, 31 chose multiple options.

c 107 participants, 56 chose multiple options and 3 chose none.

d 105 participants, 30 chose multiple options.

e *106 participants, 4 chose multiple options*.

### The Process of Decision-Making

When asked about the purpose of the IA, 92 of 106 participants chose multiple options resulting in a total of 366 answers shown in Table 3. “Protection from danger to self” was chosen most frequently (89% of the participants), followed by “solve the current emergency situation” and “treatment of the psychiatric disorder.” In about half of the PES, a patient's next of kin was actively involved in the process of decision-making. Thirty participants (30%) had contact with the patient's outpatient therapist before, during or after the PES. Of those who did not, 43 (61%) stated that the patient had no outpatient therapist or that she/he was not available, 6 (8%) were the outpatient therapists themselves, and 22 (31%) had other reasons or did not answer the question. Only 23 (21%) participants knew the patient from a present or a past treatment, 5 (5%) did so for other reasons, whereas, 79 (74%) did not know the patient prior to the consultation.

**Table 3:** Features of the decision-making process.

**Variables**	***n* (%)**
**PURPOSE OF IA**^**a**^	
Protection from danger to self	94 (89)
Solve current emergency situation	62 (58)
Treat psychiatric disorder	58 (55)
Protection from danger to others	50 (47)
Relief of social environment	33 (31)
Improve social/housing condition	26 (25)
Taking care of the patient	20 (19)
Resolve an unclear diagnosis	13 (12)
Compulsory drug treatment	4 (4)
Others	6 (6)
**THERAPEUTIC ATTITUDES**^**b**^	
Supportive	55 (51)
Directive	53 (50)
Clarifying	52 (49)
Confronting	28 (26)
Validating	24 (22)
Other or don't know	6 (6)
**ACTIVE INVOLVEMENT NEXT OF KIN**^**c**^	
Yes	54 (51)
No	51 (49)
**CONTACT OUTPATIENT THERAPIST**^**d**^	
Yes	30 (30)
No	71 (70)
**PRIOR KNOWLEDGE OF PATIENT**^**e**^	
Yes	28 (26)
No	79 (74)

IA, involuntary admission.

a 106 participants, 92 chose multiple options.

b 107 participants, 72 chose multiple options.

c 105 participants, single choice.

d 101 participants, single choice.

e *107 participants, single choice*.

Whether participants consulted with a colleague differed significantly among the subgroups of referring physicians [$\chi_{\left(4,\text{n}=105\right)}^{2}$ = 21.06, *p* < 0.001], as indicated in Table 4. Most of the non-psychiatric residents consulted with a colleague. Those who did not, felt that there was no need. Overall, about half of each subgroup felt no need for such a consultation. Nevertheless, almost 10% of both, psychiatrists and non-psychiatric senior doctors reported that a consultation would have been helpful. The use of a risk assessment tool was equally rare among all three subgroups of referring physicians. However, the rating of the potential helpfulness of such a tool differed among the subgroups [$\chi_{\left(4,\text{n}=106\right)}^{2}$ = 16.49, *p* < 0.001]. Compared to psychiatrists, both, non-psychiatric residents and senior physicians reported more frequently that the use of such a tool would have been helpful.

**Table 4:** Consultation with a colleague and use of risk assessment tool.

**Variables**	**Psychiatrist**		**OS senior**		**OS resident**		**Total**	**Chi-square**
	***n* (%)**	**sr**	***n* (%)**	**sr**	***n* (%)**	**sr**	***n* (%)**	
**CONSULTATION WITH A COLLEAGUE**^**a**^								
Took place	14 (33)	−1.2	12 (32)	−1.2	22 (85)	2.9	48 (46)	15.82^**^
Would have been helpful	4 (10)	0.7	3 (8)	0.3	0 (0)	−1.3	7 (7)	4.81
There was no need	24 (57)	0.9	22 (59)	1.0	4 (15)	−2.4	50 (48)	11.36^*^
**RISK ASSESSMENT TOOL**^**b**^								
Used	2 (5)	0.7	1 (3)	0.0	0 (0)	−0.9	3 (3)	3.12
Would have been helpful	4 (9)	−2.1	9 (24)	−0.1	14 (54)	2.9	27 (25)	17.17^**^
There was no need	37 (86)	1.1	27 (73)	0.1	12 (46)	−1.5	76 (72)	6.50

OS, other specialty; sr, standardized residual.

a 105 participants, single choice.

b 106 participants, single choice.

* p < 0.05;

** *p < 0.01*.

Most participants reported that clinical findings had contributed strongly to the decision for an IA; followed by third-party anamnesis. Other aspects contributed to a lesser extent. Details are shown in Table 5.

**Table 5:** Contribution of different aspects to the decision for IA.

**Aspect**	**Participants**^**a**^ **having chosen each category [*****n*** **(%)]**				
	**Not**	**Little**	**Int.med**.	**Strongly**	**Not app**.
Clinical findings	4 (4)	4 (4)	9 (8)	88 (83)	1 (1)
Third-party anamnesis	5 (5)	10 (9)	31 (29)	58 (54)	3 (3)
Known psychiatric diagnosis	6 (6)	16 (15)	38 (36)	37 (35)	8 (8)
Past admission(s) to psychiatric hospital	20 (19)	22 (21)	21 (20)	23 (22)	19 (18)
Past involuntary admission(s)	29 (28)	20 (19)	13 (13)	15 (15)	26 (25)
Intoxication (alcohol, drugs, medication)	29 (28)	9 (9)	12 (12)	25 (24)	29 (28)
Patient had no psychiatric outpatient treatment	29 (28)	18 (17)	11 (11)	9 (9)	37 (36)
Patient did not take prescribed medication	23 (22)	11 (11)	19 (18)	20 (19)	31 (30)

Int.med., intermediately; Not app., not applicable.

a *107 participants, missing values of all variables are 4 (4%) or below*.

## Discussion

In this study, we found that the group of physicians who mandate IAs in the canton of Zurich is very heterogenous regarding the physicians' medical specialty, level of education and experience. The consultations leading to IA took place in different locations and various parties were involved. The decision to refer patients against their will was mainly driven by clinical findings, third-party anamnesis and a known psychiatric diagnosis and served several purposes at the same time, with the protection from danger to self being named most frequently.

The key medical specialists involved in IA were psychiatrists and specialists for internal medicine. Less than 10% of participants were otherwise specialized. The internists made up for the biggest group of participants in our study, followed by psychiatrists. Looking at professional experience, more than a quarter of the participants were still residents, whereas almost half of them had more than two decades of clinical experience. The proportion of residents among psychiatric participants was much smaller and the few psychiatric residents had much more clinical experience (in years) compared to non-psychiatric participants. Clinical routine experience with IA has been discussed to elevate process quality, and the need for more specific training in the field of IA has been mentioned (30, 32, 37). Our findings suggest that training for residents working in internal medicine is likely to have a big impact on the process quality of IA in the canton of Zurich. It is reassuring that in this study a substantial part of participants has referred several patients for IA within the last 12 months. This indicates that the suggested training might find repeated opportunities for practical implementation in many cases.

We aimed at describing the PES leading to IA and found that the consultations and conducts of IAs were time-consuming, taking between 1 and 2 h in most cases. Furthermore, only about a quarter of participants had known the patient before the PES, and about half of the consultations took place in a non-medical environment, such as the patient's home or the police station. Further research is needed to find whether referring physicians can invest the time needed for IA in their clinical routine, and to what extent cutbacks in the referring process are made due to time-constraints. Prior knowledge of the patient and their medical history could shorten the referring process and has been suggested to elevate assessment quality and lengthen time of hospitalization (30). However, as the majority of the referring physicians do not know the patient from previous contacts, it was discussed that training in the handling of PES and availability of alternatives to IA should be emphasized (32). Further research is needed to better define the influence of prior knowledge and tools like psychiatric advance directives with information about the patients‘ preferences in the case of a relapse (44). Moreover, the location of the consultation may be of relevance. For instance, it was found that patients were referred for IA 3 times more often when they were assessed in a hospital emergency department or police station compared with other community locations (45), and that patients seen on a mobile crisis unit were more likely to be detained than those seen in the emergency service (29). As most studies in the field focus on a single location, limited data on how the location affects the decision to mandate IA is available. On one hand, it seems plausible that the referring physician's available options to solve a current crisis and resources to implement alternatives to IA differ according to the location of the consultation. On the other hand, it could also be that patients with a high symptom-load are more likely to be evaluated in certain locations and the named differences are hence based on patient characteristics. Given the frequency of out-of-hospital locations described in this study, future research should aim to find out what role the location plays in the process of IA. The prevalence of additional formal coercion (besides the IA itself) and the usage of informal coercion during the admission process have, to our knowledge, not been described yet. The use of informal coercion, reported in about half of cases in this study, is within the range of the prevalence in psychiatry described in a systematic review (46). The use of any form of formal coercion was reported in 25% of cases in this study. In the canton of Zurich, 6–11% of all inpatients (regardless of admission status) were found to be exposed to some form of coercion (20), and, in a recent study, it was shown that 28% of involuntary hospitalized patients experienced at least one coercive measure during the course of hospitalization (47). In conclusion, we can state that rates of coercive measures during both admission and hospitalization are comparable in the canton of Zurich. Further research has to show whether the same subgroup of patients is target to these measures in both settings.

Examining the course of the PES and the process of decision-making, we found several indications that the decision to mandate an IA of a patient might be influenced by third parties. Thus, about half of the participants actively involved a patient's next of kin in the decision-making process, whereas legally it is only requested to inform them about the decision to mandate an IA [Art. 430 Swiss Civil Code (39)]. Furthermore, most participants had contact with the outpatient therapist given there was one available and many consulted with a colleague. Also, the third-party anamnesis contributed importantly to the decision to admit the patient involuntarily. Therefore, even though a single person signs responsible for the IA, it seems to be the result of a process of integrating different views on the patient. Looking closer, we found that all participating residents either consulted with a colleague, probably their supervising physician, or felt that there was no need. This finding might be interpreted as a sign of good supervision and training, as none of the residents felt a need for a consultation but did not have the opportunity to do so. Nevertheless, almost a tenth of psychiatrists and senior doctors would have found a consultation with a colleague helpful, but for some reasons this was not possible. Our data do not give information about the reasons that forbid a consultation in these cases. Future studies should aim to evaluate if the availability of a consultation with an expert (four-eyes principle) could lower the course of a PES. The risk-assessment of danger to self or others is a crucial part in any PES. Although the use of a structured risk assessment tool was rare. This is in line with existing literature for general practitioners (42). It has been proposed that experienced physicians intuitively use similar criteria compared to such tools when assessing the risk of violence (48). Accordingly, in our study, most non-psychiatric residents, probably the group with the least experience in PES, stated that a risk assessment tool would have been helpful, whereas especially psychiatrists felt that there was no need for such a tool.

Looking at the reasons for IA, we found that almost 90% of referring physicians named protection from danger to self. This is a high proportion, compared with existing literature (42, 45, 49–51). A possible explanation might be that in Switzerland, IA is legally only possible “if the required treatment or care cannot be provided otherwise” [Art. 426 Swiss Civil Code (39)]. We can thus assume that the referring physicians find sufficient possibilities to provide treatment without IA for patients who are not at risk of harming themselves. In addition, clinical findings contributed strongest to the decision to mandate an IA, followed by third party anamnesis. This is in line with previous studies that have described the severity of symptoms and certain diagnoses as predictors for hospitalization (23, 49, 52). It is also in line with the Swiss legislation, highlighting that the clinical examination of the patient prior to an IA is obligatory and has to be conducted by the referring physicians themselves [Art. 430 Swiss Civil Code (39)]. The patient's psychiatric history, especially a known psychiatric diagnosis and, in a minority of cases, also past hospitalizations (involuntary or voluntary), contributed substantially to the decision to refer for IA. Hence on one hand, a known psychiatric diagnosis or past hospitalization could be reassuring (exert influence on.) for the referring physician, In that sense, further research should aim to gain a better understanding of the underlying grounds behind the findings that past (voluntary and/or involuntary) hospitalizations are a risk-factor for IA (53, 54). The contribution of an intoxication to the decision to refer for IA shows an interesting bimodal distribution, either contributing strongly or not at all. This could indicate that in some cases the need for IA is certain and regardless of a current intoxication, whereas in other cases only the combination of symptoms of a disorder and the intoxication leads to a condition demanding IA. One interpretation could be, that the second group of patients, in which the intoxication contributes strongly, is disallowed shortly after termination of the intoxication-symptoms. Therefore, for this subgroup of patients, another form of treatment might be more suitable than IA to a psychiatric hospital (55). Furthermore, a weak medication adherence contributed substantially to the decision to mandate an IA in about a third of cases. Discontinuation of medication has been described as a main reason to refer for IA in different countries (41, 43, 50), and a Norwegian study found treatment with neuroleptics to be the most frequently named expectation of general practitioners who referred for IA (40). Nevertheless, a meta-analysis showed that measures to enhance adherence did not significantly reduce the number of IA (56). Further research should focus on the perspective of patients who discontinued their medication and the contrasting perceived importance of medication in referring physicians.

### Strengths and Limitations

In this study we systematically collected data on three important aspects of IA: (1) the referring physician, (2) the consultations leading to IA (participants, location, duration etc.), and (3) the reasons for IA. Therefore, it gives—on a descriptive level—a broader view than studies focusing on one of these aspects (32, 38, 51). We were able to cover referrals from many different clinics and various outpatient physicians in the canton of Zurich, leading to a diverse collective of patients and referring agents. However, as we did not interview other involved parties, the view on the PES is limited to the referring physicians' perspective. Moreover, the referring physicians were invited to participate on a voluntary basis what may have biased our sample of participants. Still, comparing data on the referring physician's background in this study with data collected in another study (38) conducted in the same hospital, we can assume that our sample of participants contains no larger representation bias for the different groups of referring physicians. Data were collected only for one major university hospital in the canton of Zurich. Therefore, comparability with other regions and their respective health care structures might be limited. Due to the descriptive nature of the study, it is difficult to draw concise conclusions. Nevertheless, we think that the data can provide interesting insights and give important impulses to further research in the field.

## Conclusions

We can conclude that PES leading to IA are very heterogenous ranging from a consultative psychiatric examination on a well-equipped emergency unit of a greater hospital to a physician on his own visiting a patient in his home. Available treatment options and measures to solve a current crisis as well as patients' symptomology may vary a lot between different locations. Considering that diversity, profound training in the handling of PES seems to be indispensable to cope with the challenges that may arise during the referring process. Our data shows that especially training for residents in internal medicine could have an impact on the process of IA. Furthermore, IA has been shown to be a very time-consuming process. Further research should investigate to what extent cutbacks in the referring process are made due to time-constraints and how that affects the decision for IA. To reduce rates of IA, alternatives for patients with a high symptom load and especially for those at risk of harm to themselves are needed. Most likely, no single measure will be able to address the needs in the diverse scenarios outlined in this study.

## Data Availability Statement

The dataset generated and analyzed for this study also contains data that has not yet been analyzed and published. Therefore, the dataset is not publicly available for now. For accessibility of data, interested researchers are welcome to directly contact the authors at a later time.

## Author Contributions

SMa, MJ, and FH contributed conception and design of the study along with development of the online questionnaire. SMa acquired data, SMa and SMo performed statistical analysis and SMa wrote draft of the manuscript in close collaboration with FH. SMa, MJ, SMo, AT, ES, and FH contributed to manuscript revision, read and approved the submitted version.

### Conflict of Interest Statement

The authors declare that the research was conducted in the absence of any commercial or financial relationships that could be construed as a potential conflict of interest. The handling Editor declared a past collaboration with several of the authors MJ, ES, and FH.
